# Phylogeny and Classification of the Trapdoor Spider Genus *Myrmekiaphila*: An Integrative Approach to Evaluating Taxonomic Hypotheses

**DOI:** 10.1371/journal.pone.0012744

**Published:** 2010-09-14

**Authors:** Ashley L. Bailey, Michael S. Brewer, Brent E. Hendrixson, Jason E. Bond

**Affiliations:** 1 North Carolina Center for Biodiversity and Department of Biology, East Carolina University, Greenville, North Carolina, United States of America; 2 Department of Biology, Millsaps College, Jackson, Mississippi, United States of America; University of Edinburgh, United Kingdom

## Abstract

**Background:**

Revised by Bond and Platnick in 2007, the trapdoor spider genus *Myrmekiaphila* comprises 11 species. Species delimitation and placement within one of three species groups was based on modifications of the male copulatory device. Because a phylogeny of the group was not available these species groups might not represent monophyletic lineages; species definitions likewise were untested hypotheses. The purpose of this study is to reconstruct the phylogeny of *Myrmekiaphila* species using molecular data to formally test the delimitation of species and species-groups. We seek to refine a set of established systematic hypotheses by integrating across molecular and morphological data sets.

**Methods and Findings:**

Phylogenetic analyses comprising Bayesian searches were conducted for a mtDNA matrix composed of contiguous 12S rRNA, tRNA-val, and 16S rRNA genes and a nuclear DNA matrix comprising the *glutamyl* and *prolyl tRNA synthetase* gene each consisting of 1348 and 481 bp, respectively. Separate analyses of the mitochondrial and nuclear genome data and a concatenated data set yield *M. torreya* and *M. millerae* paraphyletic with respect to *M. coreyi* and *M. howelli* and polyphyletic *fluviatilis* and *foliata* species groups.

**Conclusions:**

Despite the perception that molecular data present a solution to a crisis in taxonomy, studies like this demonstrate the efficacy of an approach that considers data from multiple sources. A DNA barcoding approach during the species discovery process would fail to recognize at least two species (*M. coreyi* and *M. howelli*) whereas a combined approach more accurately assesses species diversity and illuminates speciation pattern and process. Concomitantly these data also demonstrate that morphological characters likewise fail in their ability to recover monophyletic species groups and result in an unnatural classification. Optimizations of these characters demonstrate a pattern of “Dollo evolution” wherein a complex character evolves only once but is lost multiple times throughout the group's history.

## Introduction

*Unfortunately, to the dismay of people seeking an immediate panacea, the molecular identification of species is fraught with the same constraints and inconsistencies that plague morphological judgments of species boundaries.[1]*

Advances in molecular biology over the past decades continue to shape the nature of systematics and taxonomy. One of the most prevalent examples of how species identification and discovery has changed is through the employment of DNA barcoding [2], an approach considered by many as a universal remedy to the “crisis” in traditional taxonomy and the only opportunity to complete an inventory of all life on the planet [3]. Briefly, DNA barcoding or DNA taxonomy is the utilization of a single gene region, in animals often the *cytochrome c oxidase I* (*coxI*) gene of the mitochondrial genome, to identify species [4]. DNA barcoding is considered by some a remedy to the idea that traditional alpha taxonomy is time intensive and is a dwindling expertise; that is, there are too few taxonomists to document earth's biodiversity within a reasonable time period.

In general, DNA barcoding or DNA taxonomy could be seen as a simplistic approach to the taxonomic enterprise. Consequently, recent attempts to refine DNA taxonomy have sought to objectively delineate species based on divergence values, the expectations of a particular diversification-extinction process, or other criteria related to gene tree–species tree construction (e.g., [5], [6]). While DNA barcoding and DNA taxonomy have detractions that would be expected of any single marker system, particularly one based on mitochondrial sequences (e.g., [7]–[10]), the insights provided by molecular sequence data have proven invaluable with respect to enhancing our understanding of speciation pattern and process [11] and distinguishing cryptic species [12]–[15]. Moreover, molecular identification of species is extensible to other disciplines within the biological sciences (e.g., species inventories, paleoecology, dietary analysis, and environmental assessments of biodiversity) [4].

Despite the advances in the field of molecular biology, the vast majority of species continue to be described on the basis of morphological features, an approach to taxonomy that has persisted for over 250 years. How species are delimited, defined, and diagnosed impacts virtually every ecological, evolutionary, phylogenetic, behavioral, physiological, comparative and conservation related study. The dwindling number of biologists doing this critical work has been attributed to a number of causes that include issues related to how taxonomic papers are cited [16], and thus their relative impact in the literature, to the perception that taxonomic constructs are not scientific hypotheses comparable to those in other areas of biology [17].

Given the importance of species discovery to all fields within the biological sciences and to addressing and assessing the global biodiversity crisis, it is surprising that the field of basic taxonomy has not flourished [18]. Rather it seems as if it has continued to decline, despite major funding initiatives like the National Science Foundation's Partnerships for Enhancing Expertise in Taxonomy [19]. One could further speculate that the promulgation of the premise that taxon-based scholars can be replaced by technicians [17] through the DNA barcoding paradigm [2] has caused irreversible harm to this already diminished field. It is not our aim here to necessarily belabor the “perils and pitfalls” of either traditional taxonomic or molecular approaches to species discovery and identification but to advocate for a balanced approach to taxonomy and classification [20]. It is clear that neither approach alone is optimal (but see caveat below) and that the field of taxonomy and its students (particularly those early in their career) only stand to gain from considering a broader perspective that entails species hypotheses that employ multiple lines of evidence. Studies that integrate both traditional approaches to taxonomy and more modern, molecular-based approaches to species delimitation clearly highlight the insights gained through a process of reciprocal illumination and serve to only further underscore the importance of monographic research.

### Mygalomorph spiders and the trapdoor spider genus *Myrmekiaphila*

The infraorder Mygalomorphae is a major lineage of spiders that includes the trapdoor spiders, funnel web spiders, tarantulas, and their kin. Despite their obvious appeal and the role they play in the stereotypical fears associated with spiders, they have long been the bane of spider systematics. Compared to its sister group, the Araneomorphae, the mygalomorph lineage comprises far fewer nominal species (2,600 vs. >38,000), but, by some estimates, this value could be much higher [21]. Contributing to their lack of attention and documented diversity are a number of factors: they live below ground and thus are difficult to collect, have retained a number of features considered primitive among spiders (e.g., simple silk-spinning apparatus, two pairs of book lungs, etc.), are relatively morphologically homogenous, and mostly lack the secondary sexual characteristics used to diagnose and distinguish the majority of spider species. As such, taxonomy of the group can be both challenging and frustrating. Moreover, mygalomorphs likewise present certain problems for molecular taxonomy and DNA barcoding. Despite the fact that major proponents of barcoding seemingly ignore this literature [22], numerous studies demonstrate that these approaches simply fail in their ability to accurately distinguish and discover species. In fact, when a standard DNA barcode distance based metric or phylogenetic species delimitation [23] is applied, virtually every population for some of these taxa [14], [21], [24] would potentially qualify as a species resulting in a gross over inflation of the group's taxonomy [12]. This phenomenon has been demonstrated for other arthropod taxa and is likely to be considerably more prevalent than previously thought, and thus confounds any strictly DNA-based or molecular phylogenetic approaches to evaluating biodiversity. Additionally, studies like the one reported herein and others demonstrate that species level paraphyly in molecular genealogies [14], [25] is common [11].

**Figure 1:**
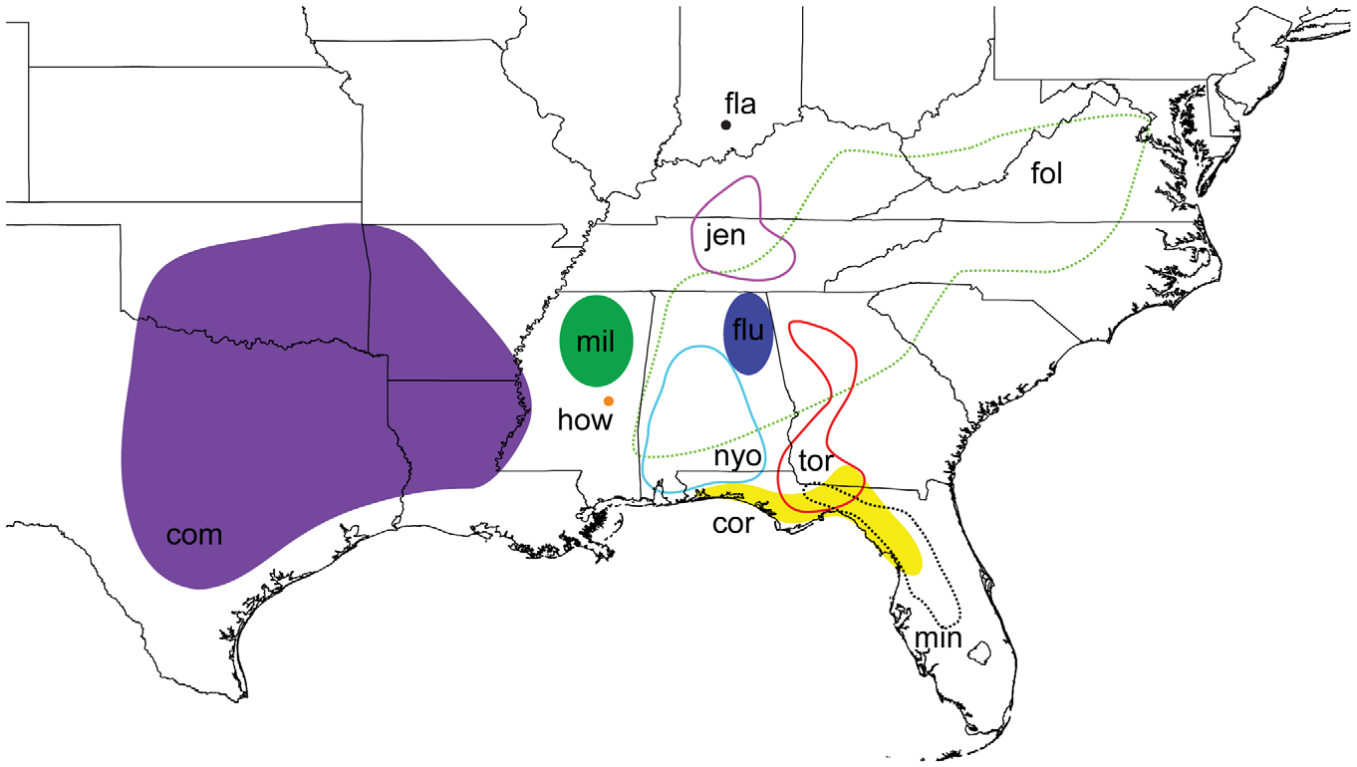
Generalized distribution map for *Myrmekiaphila* species redrawn from Bond and Platnick [28]. com – M. comstocki, cor – M. coreyi, fol – M. foliata, fla – M. flavipes, fluv – M. fluviatilis, how – M. howelli, jen – M. jenkinsi, mil – M. millerae, min – M. minuta, nyo – M. neilyoungi, tor – M. torreya.

**Figure 2:**
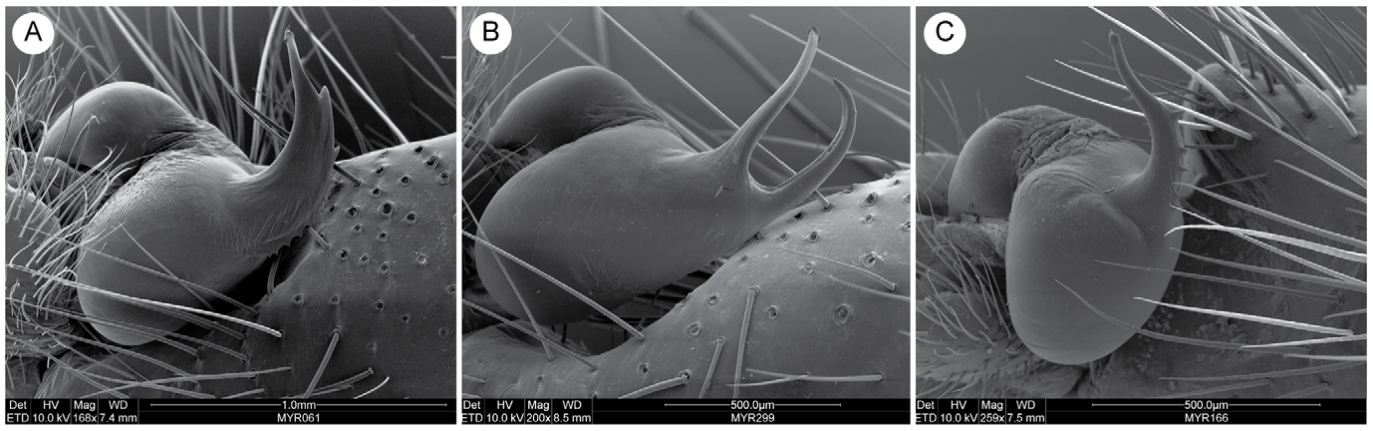
Scanning electron micrographs of exemplar *Myrmekiaphila* male palpal bulbs. A. *M. foliata*, Knox Co., Tennessee; B. *M. fluviatilis*, Marshall Co., Alabama. C. *M. minuta*, Alachua Co., Florida.

The trapdoor spider genus *Myrmekiaphila* Atkinson 1886 [26] comprises 11 closely related species that are distributed primarily throughout the southeastern United States (Figure 1). The genus is placed within the cyrtaucheniid subfamily Euctenizinae; however, the monophyly of Cyrtaucheniidae is highly contested [27]. Based on a number of phylogenetic analyses conducted across the subfamily, members of the genus appear to be sister to all of the southwestern Euctenizines save *Apomastus* Bond 2004. Like many euctenizine taxa, *Myrmekiaphila* species are relatively homogenous in general somatic morphology. However, among its closest congeners, males of each species have divergent palpal bulb morphology (Figure 2) wherein the male copulatory device is considerably more complex than in other euctenizines. Species placed within the genus are also somewhat unique in their behaviors as individuals construct burrows with side chambers that are often closed off to the main burrow by a second trapdoor [26]; most trapdoor spiders build burrows that are only sealed at the main entrance by a single door.

Until recently [28] the genus *Myrmekiaphila* had received no attention by way of a formal comprehensive taxonomic revision; only five species were described over the last 125 years, yet arachnologists had long recognized that the group contained a number of new species. The lack of attention is surprising given the relative ease at which its species can be collected and accessed. Bond and Platnick, in their 2007 revision, resolved the taxonomy of the genus and described six new species. As already discussed, species delimitation within the group was based entirely on male morphological features; that is, differences in the male copulatory apparatus and modifications to the tibia and metatarsus of the first walking leg (often termed the “mating clasper”). In some cases these differences are very subtle and require a comprehensive examination of the variation in these features across individuals, populations, and species. Females, alternatively, are much more difficult to distinguish; their somatic morphology is relatively homogenous but, in some species, there are subtle difference in spermathecae morphology. As a consequence, females present a serious issue to morphological species discovery and diagnosis.

Based exclusively on characteristics of the male palpal bulb, Bond and Platnick [28] divided the genus into three species groups. These groups were considered informal because a phylogenetic hypothesis was not available for the group at the time of the revision. The current scheme includes the *foliata* species group, which comprises three species – *M. foliata* Atkinson, *M. comstocki* Bishop and Crosby, and *M. coreyi* Bond and Platnick. Members of the *foliata* group have a male palpal bulb with a single enlarged tooth or serration but lacking a secondary prong (Figure 2A). The second species group, the *fluviatilis* group, comprises six species – *M. fluviatilis* (Hentz), *M. jenkinsi* Bond and Platnick, *M. torreya* Gertsch and Wallace, *M. neilyoungi* Bond and Platnick, *M. millerae* Bond and Platnick, *M. howelli* Bond and Platnick. All members of this group have a palpal bulb that bears a secondary accessory prong (Figure 2B). The *minuta* group comprises the single species *M. minuta* Bond and Platnick and has the simplest of palpal bulbs (Figure 2C); it lacks a secondary prong and has only a single tooth on the unbranched embolus. As mentioned, these assignments were considered informal but do infer that these major palpal structural features are likely synapomorphies for the various groups and that the more complicated branched palpal bulb has evolved only once.

The primary objectives of this study are: 1) to reconstruct the phylogeny of the genus *Myrmekiaphila* using nuclear and mitochondrial DNA sequence data; 2) based on the inferred phylogeny, evaluate the monophyly of the *foliata* and *fluviatilis* species groups; 3) to evaluate the genealogical exclusivity (i.e., evaluate species using a lineage based approach to delimitation *sensu* de Quieroz [29], [30]) of all species where population sampling was sufficient; 4) to employ the inferred molecular phylogeny to investigate the evolution of palpal bulb complexity across the genus; and 5) to develop a DNA-based framework for distinguishing among *Myrmekiaphila* species. Ultimately, the lineage-based approach we employ herein seeks to integrate morphological and molecular information into a refined taxonomic framework that can be employed to further develop the internal classification system for the genus, better delimit species, and to achieve some understanding of how genitalic characteristics have evolved across this group.

## Results

Table 1 summarizes the specimen data for taxa included as part of this study. Nine out of the 11 described *Myrmekiaphila* species were sampled (species identification is based on morphological characters described by Bond and Platnick [28]). Despite considerable effort, we were unable to collect specimens of *M. minuta* and *M. flavipes*. Our inability to find the former species is puzzling given the number of males that continue to be collected from pitfall traps in and around the type locality and suggests that the microhabitat of the species may be very different from that of the others. Alternatively, the latter species, *M. flavipes* is known only from a single specimen and thus has never again been collected since the female holotype was described in 1906. If this species does indeed represent a valid taxon (i.e., is not based on erroneous locality and/or an aberrant specimen), we are skeptical that it remains extant given our extensive sampling of mygalomorphs throughout the region. All aligned matrices and phylogenetic trees are deposited in Treebase (accession S10740).

**Table 1:** Specimens and locality information for specimens examined as part of this study.

MY_NO	*SPECIES*	LOCALITY	LAT/LONG	GenBank
MY 2025	*Myrmekiaphila fluviatilis*	AL: Madison Co., Monte Sano State Park	34.74599, −86.50653	HM122082,HM122129
MY 2034	*Myrmekiaphila fluviatilis*	AL: Lawrence Co., Borden Creek Trail	34.30959, −87.39433	HM122083,HM122130
MY 2036	*Myrmekiaphila torreya*	AL: Clarke Co., Jackson Creek on AL-69	31.59195, −87.97807	HM122084,HM122131
MY 2175	*Myrmekiaphila fluviatilis*	TN: Van Buren Co., Fall Creek Falls SP	35.66182, −85.34962	HM122085,HM122132
MY 2180	*Myrmekiaphila foliata*	TN: Campbell Co., 2.6 mi NW Rt 116 on Beech Grove Rd	36.23878, −84.19148	HM122086,HM122133
MY 2234	*Myrmekiaphila foliata*	VA: Giles Co., Cascades Rec Area	37.35383, −80.59988	HM122087,HM122134
MY 2235	*Myrmekiaphila foliata*	VA: Giles Co., Cascades Rec Area	37.35383, −80.59988	HM122088,HM122135
MY 2537	*Myrmekiaphila torreya*	AL: Butler Co., McKenzie	31.56635, −86.74021	HM122089,HM122136
MY 2538	*Myrmekiaphila torreya*	AL: Butler Co., McKenzie	31.56635, −86.74021	HM122090,HM122137
MY 2539	*Myrmekiaphila torreya*	AL: Butler Co., McKenzie	31.56635, −86.74021	HM122091,HM122138
MY 2540	*Myrmekiaphila torreya*	AL: Butler Co., McKenzie	31.56635, −86.74021	HM122092,HM122139
MY 2548	*Myrmekiaphila torreya*	AL: Baldwin Co., Hurricane Landing	30.81922, −87.91383	HM122093,HM122140
MY 2551	*Myrmekiaphila torreya*	FL: Santa Rosa Co., Escambia River	30.95616, −87.21464	HM122094,HM122141
MY 2552	*Myrmekiaphila torreya*	FL: Santa Rosa Co., Escambia River	30.95616, −87.21464	HM122095,HM122142
MY 2556	*Myrmekiaphila torreya*	FL: Santa Rosa Co., Escambia River	30.95616, −87.21464	HM122096,HM122143
MY 2557	*Myrmekiaphila coreyi*	FL: Washington Co., nr. FL-20 on FL-79	30.46376, −85.86335	HM122097,HM122144
MY 2558	*Myrmekiaphila coreyi*	FL: Washington Co., nr. FL-20 on FL-79	30.46376, −85.86335	HM122098,HM122145
MY 2559	*Myrmekiaphila coreyi*	FL: Washington Co., nr. FL-20 on FL-79	30.46376, −85.86335	HM122099,HM122146
MY 2561	*Myrmekiaphila coreyi*	FL: Washington Co., nr. FL-20 on FL-79	30.46376, −85.86335	HM122100,HM122147
MY 2562	*Myrmekiaphila coreyi*	FL: Washington Co., nr. FL-20 on FL-79	30.46376, −85.86335	HM122101,HM122148
MY 2568	*Myrmekiaphila torreya*	FL: Liberty Co., Apalachicola River	30.43181, −84.99387	HM122102,HM122149
MY 2569	*Myrmekiaphila torreya*	FL: Liberty Co., Apalachicola River	30.43181, −84.99387	HM122103,HM122150
MY 2570	*Myrmekiaphila torreya*	FL: Liberty Co., Apalachicola River	30.43181, −84.99387	HM122104,HM122151
MY 2571	*Myrmekiaphila torreya*	FL: Liberty Co., Apalachicola River	30.43181, −84.99387	HM122105,HM122152
MY 2576	*Myrmekiaphila torreya*	FL: Liberty Co., nr. Sweetwater	30.51075, −84.95982	HM122106,HM122153
MY 2715	*Myrmekiaphila fluviatilis*	TN: Sequatchie Co., 2.6 mi NW TN-28 on Fredonia Rd	35.39514, −85.39662	HM122107,HM122154
MY 2801	*Myrmekiaphila fluviatilis*	TN: Lawrence Co., David Crockett SP	35.26252, −87.36217	HM122108,HM122155
MY 2836	*Myrmekiaphila jenkinsi*	KY: Edmonson Co., Collie Ridge Trail	37.25555, −86.15842	HM122109,HM122156
MY 3387	*Mymekiaphila comstocki*	AR: Polk Co., Caney Creek WMA	34.42985, −94.13922	HM122110,HM122157
MY 3388	*Mymekiaphila comstocki*	AR: Polk Co., Caney Creek WMA	34.42985, −94.13922	HM122111,HM122158
MY 3389	*Mymekiaphila comstocki*	AR: Polk Co., Caney Creek WMA	34.42985, −94.13922	HM122112,HM122159
MY 3582	*Myrmekiaphila torreya*	FL: Liberty Co., Torreya State Park	30.56971, −84.95095	HM122113,HM122160
MY3590	*Myrmekiaphila torreya*	AL: Butler Co., Persimmon Creek	31.56675, −86.73998	HM122114,HM122161
MY 3593	*Myrmekiaphila torreya*	AL: Butler Co., Persimmon Creek	31.56675, −86.73998	HM122115,HM122162
MY 3594	*Myrmekiaphila torreya*	AL: Butler Co., Persimmon Creek	31.56675, −86.73998	HM122116,HM122163
MY 3595	*Myrmekiaphila howelli*	MS: Newton Co., Hwy 494, Chunky-Duffee Rd	32.50092, −90.01086	HM122117,HM122164
MY 3597	*Myrmekiaphila millerae*	MS: Grenada Co., Scott Rd, Duncan Rd.	33.72371, −90.01086	HM122118,HM122165
MY 3598	*Myrmekiaphila millerae*	MS: Grenada Co., Scott Rd, Duncan Rd.	33.72371, −90.01086	HM122119,HM122166
MY 3601	*Myrmekiaphila millerae*	MS: Choctaw Co., Choctaw WMA, Campground Hiking Trail, near Hwy 15	33.27334, −89.14489	HM122120,HM122167
MY 3602	*Myrmekiaphila millerae*	MS: Choctaw Co., Choctaw WMA, Campground Hiking Trail, near Hwy 15	33.27334, −89.14489	HM122121,HM122168
MY 3603	*Myrmekiaphila millerae*	MS: Choctaw Co., Choctaw WMA, Campground Hiking Trail, near Hwy 15	33.27334, −89.14489	HM122122,HM122169
MY 3605	*Myrmekiaphila neilyoungi*	AL: Shelby Co., Birmingham, Shades Ck	33.46510, −86.78112	HM122123,HM122170
MY 3606	*Myrmekiaphila torreya*	AL: Shelby Co., Birmingham, Shades Ck	33.46510, −86.78112	HM122124,HM122171
MY 3607	*Myrmekiaphila neilyoungi*	AL: Shelby Co., Birmingham, Shades Ck	33.46510, −86.78112	HM122125,HM122172
MY 3611	*Myrmekiaphila fluviatilis*	AL: Jackson Co., Scottsboro near the West side of Tennessee River, Hwy 35	34.64560, −85.98534	HM122126,HM122173
MY 0736	*Promyrmekiaphila* sp.	CA: Glenn Co., hwy 162, 0.9 mi East of Stony Creek Crossing	39.15550, −122.51330	HM122080,HM122128
MY2595	*Aptostichus* sp.	CA: Riverside Co., Winchester, Leona rd ∼1.0 mi South of intersection Patton Ave.	33.67712, −117.11578	HM122081,HM122127

GeneBank Accession numbers reference the 12S–16S and 192fin data respectively.

### Summary of sequence data

Approximately 1348 base pairs (bp) of the mitochondrial 12S/16S rRNA gene (including the short interveining tRNA-VAL gene) and 481 bp of *glutamyl- & prolyl-tRNA synthetase* (192fin) nuclear protein coding region were sequenced from the majority of specimens (Table 1, GenBank accession numbers HM122080-HM122173). Primer fidelity across taxa was not always consistent; consequently, some specimens had truncated sequence length for the 12S/16S rRNA gene. Base compositions were as follows: 12S/16S (A = 0.38938, C = 0.13952, G = 0.14214, T = 0.32895) and 192fin (A = 0.23891, C = 0.26716, G = 0.21862, T = 0.27531). In PAUP* [31], a χ^2^ test of homogeneity of base frequencies across taxa found that the sequences were not significantly heterogeneous for 12S/16S (χ^2^ = 46.357, df = 129, P>0.05) or 192fin (χ^2^ = 4.895, df = 138, P>0.05).

### Phylogenetic Analyses

The models of DNA substitution obtained from Kakusan 3 for each partition were: 12S (GTR+G), tRNA-VAL (HKY85+G), 16S (HKY85+G), 192fin position 1 (F81+G), 192fin position 2 (JC69), and 192fin position 3 (K80+G). The harmonic means for all post burn-in topologies were 12S/16S (−10,815.18), 192fin (−1,425.97), and combined −12,231.79); arithmetic means were 12S/16S (−10,762.54), 192fin (−1,377.86), and combined (−12,231.80). The number of trees sampled from the 95% credible set were as follows: 12S/16S (81,700), 192fin (28,499), and combined (100,763).

**Figure 3:**
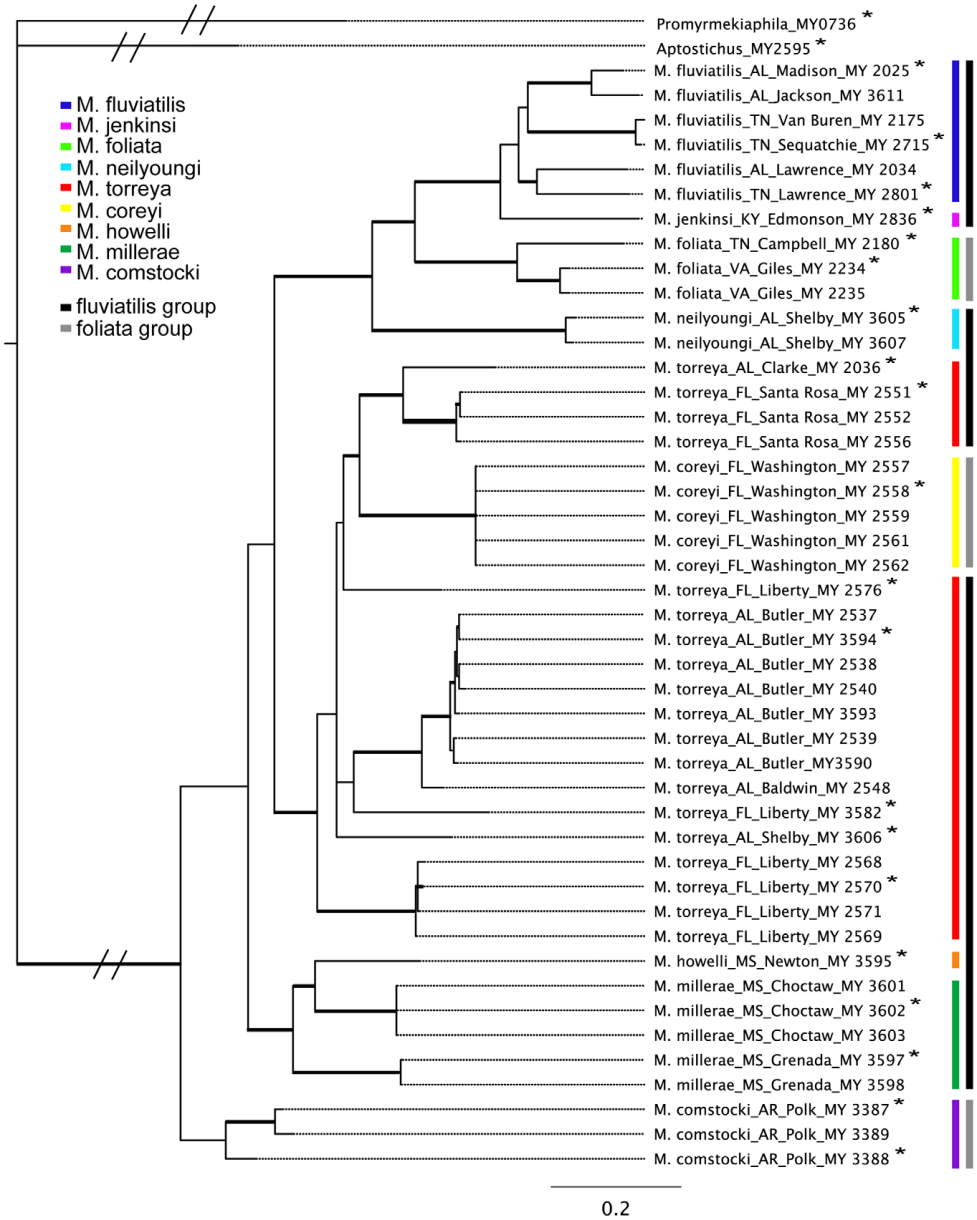
Preferred tree topology based on Bayesian concatenated analysis of 12S/16S mtDNA genes and the nuclear protein coding glutamyl- & prolyl-tRNA synthetase gene. Key (inset) references species and species groups defined by Bond and Platnick (2007). Thickened branches indicated posterior probabilities >95%.

The recovered topologies for individual genes were highly concordant, but the 192fin tree lacked resolution at intermediate levels (available in Treebase accession S10740). In both single gene analyses and the concatenated analysis, most nominal species were recovered as genealogically exclusive with relatively high support (concatenated posterior probability (pp)  = 0.88 *M*. *fluviatilis*; pp = 1.00 other monophyletic species; Figure 3) with the following exceptions: *M*. *foliata* was polyphyletic in the 192fin tree and *M*. *torreya* was paraphyletic with respect to *M*. *coreyi* in the 12S/16S, 192fin, and concatenated trees. *Myrmekiaphila millerae* was paraphyletic with respect to *M*. *howelli* for both data sests. *Myrmekiaphila comstocki* was basal but paraphyletic in the 12S/16S tree and monophyletic but nested further up within the tree in the 192fin analysis. The concatenated analysis recovered *M*. *comstocki* as basal and monophyletic but with low support (pp = 0.69). The species group assignments [28] were not recovered in any of the trees.

### Bayes factor assessment of taxon monophyly and ancestral state reconstruction

To evaluate whether the species groups defined by Bond and Platnick [28] were plausible alternatives to the topology recovered in the concatenated tree (Figure 3), we ran a separate analysis that constrained the monophyly of the *foliata* group (*M*. *foliata*, *M*. *comstocki*, and *M*. *coreyi*). This analysis was run for 2,500,000 generations with the first 625,000 discarded as burnin. The resulting tree's harmonic mean of the -log likelihood values was 12,340.13. The Bayes Factor value indicates that the constrained tree is 108.34 greater than the unconstrained tree; values ≥10 are considered strong evidence that the tree topologies are not similar. Based on these results, the *foliata* and *fluviatilis* species groups are unequivocally polyphyletic for these data.

To assess whether a monophyletic *M*. *torreya* is a plausible alternative to being paraphyletic with respect to *M*. *coreyi*, we performed a second analysis constraining *M*. *torreya* monophyly. This analysis was run for 3,000,000 generations (first 750,000 discarded as burnin). The constrained topology harmonic mean -log likelihood value was 12,281.25, resulting in a Bayes Factor of 49.46. The unconstrained tree that recovers *M. torreya* paraphyly is thus the more strongly supported hypothesis given the data.

**Figure 4:**
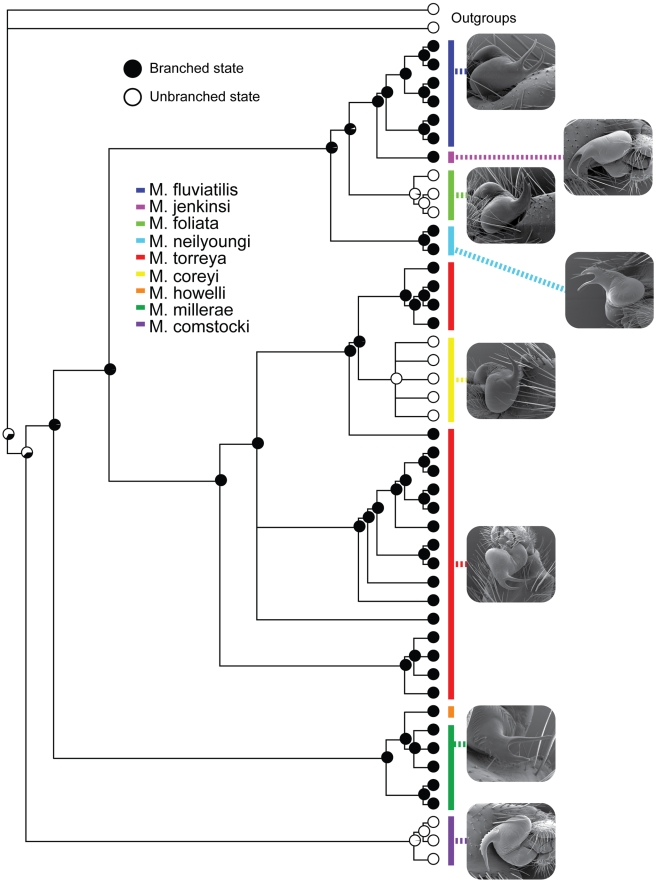
Ancestral state reconstructions for unbranched vs. branched embolus. Pie diagrams indicate probability of observing a particular state.

The divided versus undivided embolus character system that was used to assign taxa to species groups (depicted in Figure 2) is shown to be evolutionarily uninformative for this purpose. The outgroups used in this analysis and all other known euctenizine taxa [32], [33] have the undivided state, thus the divided character state appears to have evolved shortly after the splitting of the lineage that gave rise to *M*. *comstocki* (the sister group to all other *Myrmekiaphila* species). Consequently, the clade that comprises the rest of the genus (*sans M. comstocki*) has the divided state as the ancestral optimization. Reversals to the undivided state occur twice across for the phylogeny, independently in the *M*. *torreya* and *M*. *foliata* lineages (Figure 4).

## Discussion

*“Are species epistemologically the basis of phylogenetic analysis or the result of it?” [34]*

The combined analysis of the mitochondrial and nuclear protein coding molecular data sets present an interesting, but not necessarily uncommon, juxtaposition of morphological, DNA-based, or lineage-based approaches to species delineation, the delimitation of species groups, and interpretations of how genitalic morphology evolves. In particular, these results show that neither approach, taken alone, is entirely sufficient and that an integrative view to taxonomy and classification is likely to present the more comprehensive view of species boundaries, phylogeny, and of evolutionary processes.

Alpha taxonomy in spiders is typically approached from a morphological perspective that is based primarily on differences in genitalic structures [35]. The generally accepted paradigm for spiders [34] and other arthropod groups (e.g. Diplopoda [36]) is that genitalic features evolve rapidly in concert with speciation as a function of sexual selection by female choice and/or sexual conflict (SSFC-SC) [37], [38]; however, see Bond et al. [36] for the “risks” associated with this assumption. Mygalomorph spiders, as discussed earlier, typically lack many of the diagnostic features found in their more diverse sister taxon the Araneomorphae and in particular lack complex genitalia. The species of which the genus *Myrmekiaphia* is composed are somewhat unique among other mygalomorph taxa in that male palpal features actually vary interspecifically. For this reason this study, like that of Astrin et al. [35], provides a generally straightforward case wherein species appear to be morphologically unambiguous and as such provide a framework that can be used to assess the efficacy of molecular data to recover species defined on the basis of genitalic differences.

### Genitalic evolution and related biogeography

**Figure 5:**
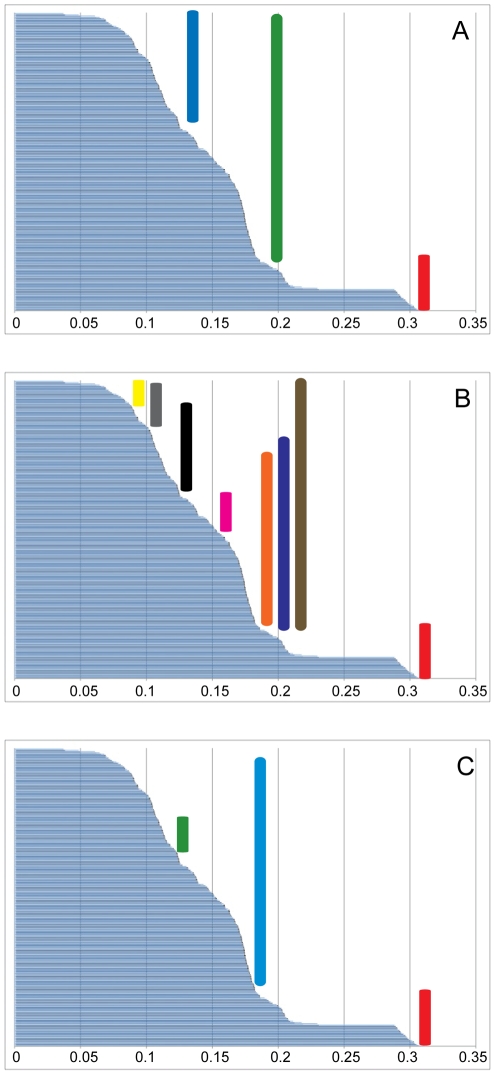
Distribution of pairwise distances for taxa representing breadth of divergence for each clade and/or species. Taxa used in this analysis are indicated by an asterisks in Figure 3. A. RED  =  outgroup, GREEN  =  interspecies, BLUE  =  intraspecies; B. RED  =  outgroup, BROWN  =  between species group, PURPLE  =  *fluviatilis* group to *torreya* group, ORANGE  =  *fluviatilis* group to *howelli* group, PINK  =  *torreya* group to *howelli* group, BLACK  =  *fluviatilis* group to *comstocki* group, GREY  =  *torreya* group to *comstocki* group, YELLOW  =  *howelli* group to *comstocki* group; C. RED:  =  Outgroup, BLUE  =  *M. coreyi* to non-*torreya* group species, GREEN =  *M. coreyi* to *M. torreya*.

The optimization of the genitalic characters on the preferred tree topology (Figure 4) shows a somewhat unexpected pattern of change in palpal bulb morphology. Not surprisingly, the unbranched condition is optimized at the root of the phylogeny with a shift to the branched condition in the daughter node above the lineage that includes all *Myrmekiaphila* species. It should be noted here that the unbranched state is somewhat of an oversimplification. Although the plesiomorphic condition is unbranched this state is further modified in *Myrmekiaphila* to include serrations as illustrated in Figure 2. Once gained the branched state is then lost in two independent lineages further up the tree. Most notable is the loss of the branched state within the clade comprising populations of *M. torreya* and *M. coreyi* (see discussion of species paraphyly below). Figure 5 illustrates the distribution of pairwise sequence distances in the 12S/16S data among exemplar lineages representing the breadth of phylogenetic diversity among all *Myrmekiaphila* species. *Myrmekiaphila coreyi* has pairwise divergence values for the mtDNA loci that are well within the range of intraspecific sequence divergence estimated among the other species thus the considerable morphological change in the *M. coreyi* lineage is not reflected in the molecular data.

Although somewhat anecdotal the pairwise distance results (summarized in Figure 5) suggest that genitalic evolution may have outpaced the rate of divergence observed in the molecular data thus lending support to a SSFC-SC hypothesis to explain these differences in palpal bulb structure. Bond et al. [36] outlined three patterns of genitalic/molecular divergence evolution, the first one being a pattern comparable to what we see in *M. coreyi* in which the species' morphology has sorted ahead of the “neutral” molecular marker. We are more likely to infer SSFC-SC for *M. coreyi* as opposed to any of the other species given the relatively short branch lengths on which these individuals occur. Although genitalic complexity and divergence is generally widespread throughout the genus, the remaining species are sorted in their morphology and molecules. That said, divergence across the genus is relatively shallow and some nodes lack strong support. Consequently, a conservative conclusion would be that there is insufficient data available to support or reject a hypothesis of SSFC-SC (pattern 2; [36]) for the other taxa.

The observed geographic ranges for *Myrmekiaphila* species show some interesting patterns that seem to be correlated with genitalic morphology (Figures 1 and 4). In all cases, species are found in sympatry (i.e., with overlapping geographic ranges) only with congeners that have the other male palpal bulb character state (divided versus undivided). Moreover, in the only instance where three species are sympatric (*M. torreya, M. coreyi*, *M. minuta*), a third distinctive genital morphology is observed; *M*. *minuta* has a unique palpal bulb morphology and thus was assigned to its own species group based on this characteristic [28]. However, as already discussed for the other species groups, such an assignment may not represent a natural grouping given the plasticity in genitalic morphology across the genus.

Although sampling efforts may not be extensive enough to draw definitive conclusions, the geographic patterns in genital morphology throughout the genus are noteworthy. First, the closest relatives of *Myrmekiaphila* are found in the American Southwest and Mexico [32]. Consequently, we hypothesize that populations from these regions expanded eastward into the southeastern United States. Such a hypothesis seems reasonable because *M*. *comstocki* has the “ancestral”, unbranched palpal bulb character state, is distributed furthest to the west, and is the basal-most species (i.e., is sister to the remaining species in the genus). Subsequent to this expansion, the more complex genital morphology appears to have evolved along the parent node to the remaining species. The *M*. *millerae* and *M*. *howelli* lineages are distributed to the east of the Mississippi River in north and central Mississippi, respectively. The remaining two clades (*M. torreya* and *M. coreyi*; *M. fluviatilis*, *M. jenkinsi*, *M. foliata*, and *M. neilyoungi*) appear to have diverged and subsequently expanded their ranges into the Southern Coastal Plain of the Gulf of Mexico with further expansion eastward and northward. The ranges of the currently recognized species in these clades are suggestive of classic allopatric speciation with two striking exceptions. *Myrmekiaphila coreyi* is monophyletic but nested within a paraphyletic *M*. *torreya* (see discussion below) and has undergone a character state reversal to the primitive unbranched palpal condition. Likewise, *M*. *foliata* has also reverted to the ancestral, unbranched state independently. The reversal to the unbranched condition in *M. foliata* and *M. coreyi* may be features that have allowed them to co-occur with other closely related lineages (Figures 1 and 4). While the phylogenetic placement of *M*. *minuta* is unknown it seems reasonable to hypothesize that its uniquely divergent morphology (general somatic and genitalic) has likely served to isolate the species in sympatry from *M. coreyi* and *M*. *torreya*.

The loss of the more complex mating system in *M. coreyi, M. minuta*, and *M. foliata* may have facilitated the expansion of these species' ranges by reinforcing prezygotic barriers to mating in sympatry. Reinforcement has been demonstrated at the inter- and intraspecific levels in a number of classic studies [39]–[44] and in more recent examples where sympatric species seem to have evolved differences in mating morphology as a consequence of character displacement [45], [46]. Greater genitalic divergences in both male penis length and female vagina length were found in sympatric populations of *Satsuma* snails irrespective of environmental, genetic, or geographic effects [45]. Sympatric *Parafontaria* millipede species that lack effective precopulatory isolation were shown to have developed mechanical isolation by means of gonopod and cyphopod sexual morphological character displacement that effectively prevented interspecific transfer of the spermatophore to sympatric females [46].

Finally, it appears that the evolution of genitalic complexity (the branched embolus character state) follows a pattern of Dollo's Law (see Collin & Miglietta [47] and Goldberg & Igic [48] for recent reviews). That is, once lost a more complex character can never be regained. Given the extent of the occurrence of losses (only twice) within the genus and out towards the tips of the inferred phylogeny, it is probably more precise to infer that a reversal to the more “simple” plesiomorphic condition seems to be an evolutionary transition that can occur with relative ease. Although we are confident in our assignment of the character state and its frequency at the root node (see Goldberg & Igic [48] for major causes of errors when examining reversals), our sampling across the phylogeny is incomplete and thus the addition of more taxa could have an impact on the optimization of this character (namely, we were unable to include *M. minuta* and do not know how its position in the phylogeny and its simpler genitalia would affect our interpretation of these changes).

### Species and species group delimitation

As already outlined above, genitalic characters are the feature *de rigueur* for the vast majority of spider taxonomic studies; the revision of *Myrmekiphila* by Bond and Platnick [28] was no exception. At taxonomic levels above species it remains relatively clear that genitalic features have limited utility [34] but see Song and Bucheli [49]. Consequently, the species groups delineated by Bond and Platnick [28] on the basis of branched vs. unbranched embolus appear to be unnatural groups with respect to the inferred molecular phylogeny. The ease at which this seemingly complex feature can be lost in parallel across the group's history provides a cautionary note regarding the use of these features in phylogenetic analyses. This is particularly likely to hold true for groups where there is a paucity of other non-genitalic somatic features (i.e., some large percentage of the morphological character matrix is derived from genitalia).

With respect to the molecular data, the species hypotheses put forth by Bond and Platnick [28] are also in conflict. Two species, *M. torreya* and *M. millerae*, are paraphyletic with respect to *M. coreyi* and *M. howelli*, respectively. Subsequent review, by us, of the specimens examined by Bond and Platnick [28] confirm that these species are diagnosable and indeed do not appear to overlap morphologically. *Myrmekiaphila coreyi* has a palpal bulb morphology that is discretely different from *M. torreya* (unbranched vs. branched embolus, discussed in detail above), has divergent mating clasper morphology, and is considerably smaller in size. The differences in palpal bulb and mating clasper morphology between *M. millerae* and *M. howelli* are subtler, however, female spermathecae morphology of the latter is considerably different from that of *M. millerae* (note: the female of *M. howelli* was collected and examined as part of this study for the first time). Furthermore, the distributions of these two species in Mississippi do not overlap. Based on these observations we remain confident in the species delimitations put forth in the taxonomic revision [28], however, this does present some problems given the inferred evolutionary history of the group (Figure 3).

As reviewed by Funk and Omland [11], species paraphyly is more prevalent than previously thought, occurring in approximately one out of every five species surveyed. Likewise, a number of studies to date that have focused on species boundaries in other mygalomorph taxa have uncovered species non-exclusivity [14], [24], [50] further suggesting that it is actually quite common. As discussed above, we discard the supposition that these data falsify the species hypotheses for *M. coreyi* and *M. howelli*. However, the species hypotheses with respect to the composition of *M. torreya* and *M. millerae* do require further examination. One obvious alternative is that these lineages comprise a set of cryptic species. Without question, one of the principal outcomes stemming from the assessment of species boundaries in light of DNA sequence data is the prevalence of morphologically indistinguishable lineages that are allopatric and likely reproductively isolated and thus qualify as cryptic sibling species [51]–[54]. First, it is our hypothesis that *M. torreya* lineage does not comprise cryptic species. Elevating all *M. torreya* lineages that are at comparable phylogenetic levels to *M. coreyi* would result in five additional species. Given the relatively shallow levels of divergence across these lineages in both the mitochondrial and nuclear data sets and the general lack of geographical concordance throughout the *M. torreya* clade, it is our opinion that such a hierarchical driven recalibration of species boundaries would be flawed. However, sampling of additional nuclear markers (e.g., microsatellites) to quantitatively assess population parameters like gene flow would be necessary to fully test the hypothesis that *M. torreya* comprises a single cohesive species. That said, the general lack of geographical concordance within these six lineages is consistent with a hypothesis of recent gene flow across populations indicating that a sufficient period has not elapsed to sort ancestral polymorphisms. And, while a similar recalibration would not have the same drastic effect on the *M. millerae* lineages (only one additional species need be recognized), the geographical sampling across this species is insufficient to warrant additional nominal species. For the time being it would seem justified to retain *M. millerae* and *M. torreya* as paraphyletic species as these hypotheses embrace the budding nature of speciation and recognize the potentially rapid rate at which genitalia can evolve as a consequence of SSFC-SC (see discussion above). We have generally applied the logic for species delimitation outlined by Bond and Stockman [14] and Wiens and Penkrot [23] that weigh geographical concordance among lineages as a first test of cryptic species boundaries.

Our results further exemplify the shortcomings of taking an exclusively molecular or lineage-based approach to species delimitation. Although the mtDNA gene sequences analyzed for this study are not from the barcoding region (i.e., *coxI*), 12S/16S data have proven to be an effective marker for species level studies in a number of spider groups [14], [21], [24], [55]–[57] and may be the superior marker (relatively speaking) for spiders [35] and other taxa (e.g., corals [58]). Nevertheless, the same caveats apply to species constructs based on these markers (albeit linked as part of the mitochondrial “gene” to the barcoding region). With regards to DNA barcoding/taxonomy for *Myrmekiaphila* species, a few general observations can be made concerning the adequacy of these data:

1. The 12S/16S sequences employed are inadequate for species discovery within the genus. Given the degree of paraphyly observed in these analyses (for at least two of the species), species boundaries based on a lineage/phylogenetic or DNA-profile approach fail to recognize all of the species that comprise this genus.
2. As has been already demonstrated in other metazoan taxa [59] there is unequivocally no barcoding “gap” in these data (Figure 5). That is, it is not possible to formulate a metric of DNA divergence for this group that would consistently recover species boundaries. Varying rates of molecular and morphological evolution within this group obfuscate any such signal in these data. Such an observation is not endemic to this group.
3. To our knowledge, this study represents the first adaptation of the *glutymyl-* and *prolyl tRNA synthetase* (192fin) nuclear protein-coding gene for species level phylogenetic analyses; previous analyses used this gene for deeper levels within arthropod phylogeny [60]. As expected, it was less likely to resolve shallow branches in our tree at the population/species interface. However, it did provide useful signal at more intermediate levels within the phylogeny. Within other mygalomorphs for which deeper divergence is expected across populations and species (e.g., *Aptostichus* [14], *Antrodiaetus* [54]), this marker may provide an alternative to rRNA genes commonly used in phylogeographic studies (e.g., 28S, 18S, and ITS).
4. Despite the obvious shortcomings in these data, the 12S/16S mtDNA sequences can be employed in the diagnosis and subsequent identification of species. As we document in the taxonomy section below, DNA diagnoses can be formulated for the species included as part of this study. Consequently, rapid identification of species, regardless of life stage, is possible.

### Conclusions

This study highlights the need for an integrative and iterative approach to species delimitation and further makes the point that molecular data are insufficient when interpreted alone. Furthermore it exemplifies the contributions of morphology and biogeography to addressing questions not only related to delineating species but to investigating evolutionary questions like sexual selection and reinforcement; questions that *cannot* be effectively addressed by molecules alone. As DNA data have become more common for investigations of species boundaries, so too have the prevalence of morphologically cryptic species. Although our “discovery” of species paraphyly is not uncommon [11], we submit that such instances demonstrate that species crypsis is not a phenomenon to which only morphological data are prone. Paraphyly, or non-exclusivity, will disguise species diversity in pairwise divergence gap analyses and in phylogenetic/neighbor-joining profiles much in the same way that morphological stasis would in an analysis that relies on phenotypic characters. From an evolutionary perspective, morphological crypsis manifests itself as a lack of precision in the data (more inclusive groupings). Alternatively, the issues in the molecular data (species polyphyly), at least within the context of this analysis, seem to be more a problem of both precision and accuracy. Consequently, it seems clear that neither a traditional morphological approach nor a molecular approach to taxonomy is always going to adequately recover all species diversity; multiple sources of data are necessary to accurately and precisely recover diversity [61]. Because morphological revisions of taxa present a more complete and accurate, albeit potentially less precise, picture of the taxa summarized, it is our opinion that efforts to shift taxonomy away from revisionary studies to molecular-based barcoding studies are foolhardy and largely uninformed [1], [62]. As so elegantly discussed by Meier [63], the “real frontiers in taxonomy” are in developing ways to integrate data across multiple sources (DNA, morphology, behavior, and ecology).

### Taxonomic Summary

**Figure 6:**
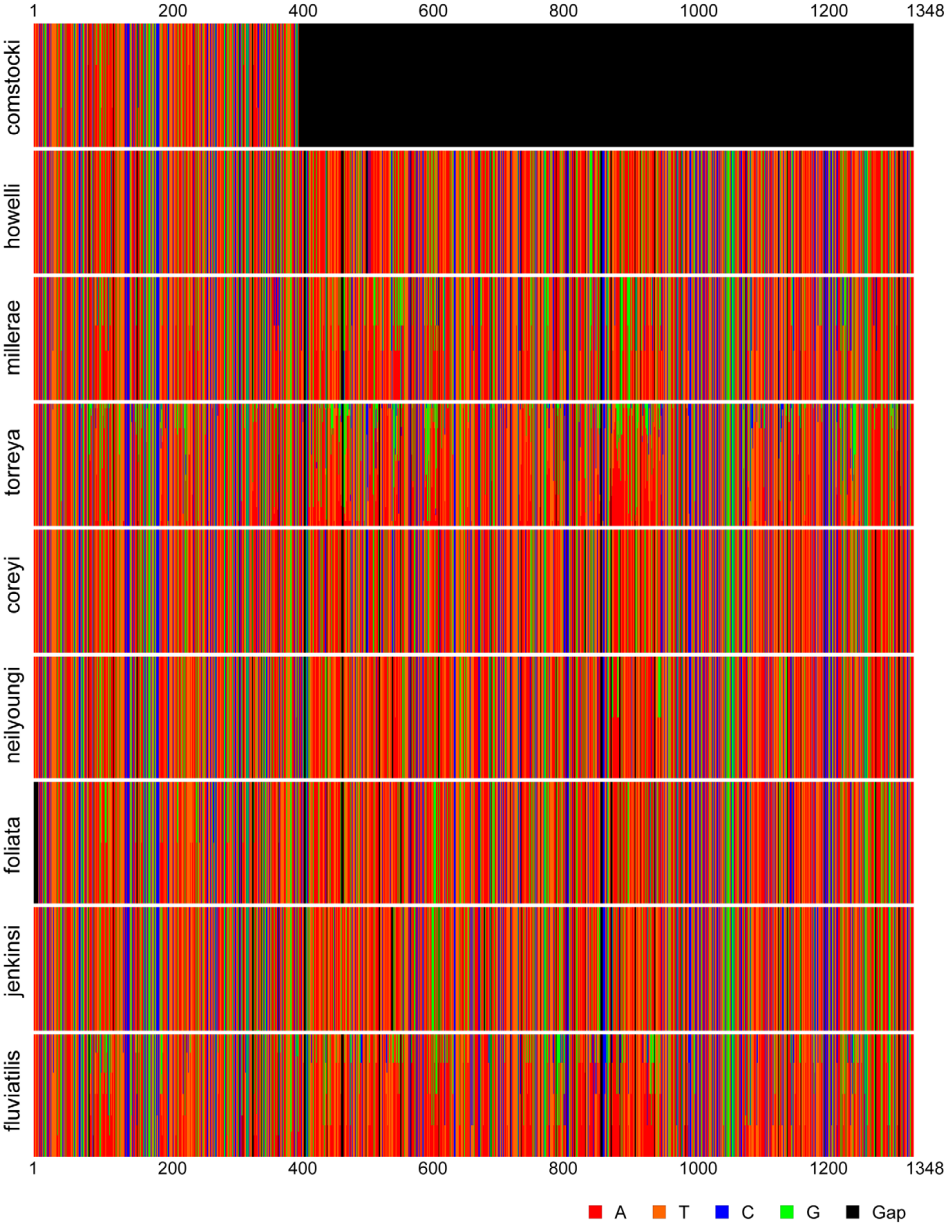
Graphical representation of “DNA fingerprint” for *Myrmekiaphila* species sequenced as part of this study.

We summarize below the revised systematics of *Myrmekiaphila* based on the phylogenetic hypothesis inferred from the molecular data. Zoobank (http://zoobank.org) LSID assignments made since the revision by Bond and Platnick [28] and molecular diagnoses for all species are also summarized here. The latter (using 12S/16S mtDNA) are inferred on the basis of ancestral nucleotide states reconstructed using parsimony implemented in the computer program MacClade [64]. The unique combination of characters states listed include changes that are unique and uniform above the node defining a species (listed in *italics*) and characters that are uniform above the node but may have the same state elsewhere on the tree. For the two instances involving species paraphyly the substitutions in bold reference the reconstruction for those sites that are uniquely derived further up the tree for the “embedded” species. The unique combination character formulations follow the approach and justification outlined by Bond [24]. Position numbers refer to the column in the matrix accessioned in Treebase (S10740). Variation in the mtDNA region is summarized graphically (Figure 6) using the online interface for the computer program Fingerprint [65].

Family Cyrtaucheniidae Simon, 1892

Subfamily Euctenizinae Raven, 1985

Genus *Myrmekiaphila* Atkinson, 1886

### *The* comstocki *species group*

***Myrmekiaphila comstocki*** **Bishop and Crosby**

**urn:lsid:zoobank.org:act:48049DAB-D05C-4E73-A804-EB97C57D4C21**

*Myrmekiaphila comstocki* Bishop and Crosby, 1926: 168,169; *Myrmekiophila fluviatilis* (misidentification): Petrunkevitch, 1929: 516; *Myrmekiaphila comstocki* Gertsch, 1935: 3; *Myrmekiaphila comstocki* Bond and Platnick, 2007: 11–13.

*Molecular diagnosis. Myrmekiaphila comstocki* can be diagnosed on the basis of the following a single unique 12S/16S nucleotide substitution: G (108). Visual profile of sequence variation is summarized in Figure 6.

### *The* millerae *species group*

***Myrmekiaphila millerae*** **Bond and Platnick**

**urn:lsid:zoobank.org:act:2B317FB8-CB36-4730-803B-38B6100DBEBB**

*Myrmekiaphia millerae* Bond and Platnick, 2007: 24–26.

*Molecular diagnosis. Myrmekiaphila millerae* can be diagnosed on the basis of the following unique combination of 12S/16S nucleotide substitutions: **A (108**), **T (120)**, A (152), A (161), **A (265)**, A (658), A (659), **T (668)**, A (673), **A (795)**, G (866), T (892), **A (895)**, G (902), **G (904)**, **A (938)**, T (955), **A (966)**, A (967), **A (1037)**, G (1123), C (1177). Visual profile of sequence variation is summarized in Figure 6.

***Myrmekiaphila howelli*** **Bond and Platnick**

**urn:lsid:zoobank.org:act:7AB0F182-493C-4378-B10C-26A9B6F717E9**

*Myrmekiaphila howelli* Bond and Platnick, 2007: 16–19.

*Molecular diagnosis. Myrmekiaphila howelli* can be diagnosed on the basis of the following unique combination of 12S/16S nucleotide substitutions: A (84), G (89), T (97), *T (108)*, G (119), *C (120)*, C (196), *G (265)*, G (275), G (435), T (468), G (497), G (503), C (514), C (517), T (523), T (560), A (602), G (607), A (609), T (612), G (613), A (631), *C (668)*, A (691), G (694), A (789), C (793), *G (795)*, A (816), A (847), G (858), T (885), *T (895)*, *T (904)*, G (907), A (908, 912, 915, 916, 927), *G (938)*, G (944), T (958), G (959), *T (966)*, G (1016), *G (1037)*, G (1119), T (1175), *A (1245)*, T (1250), A (1259), T (1262), *T (1319)*, T (1321). Visual profile of sequence variation is summarized in Figure 6.

### *The* torreya *species group*

***Myrmekiaphila torreya*** **Gertsch and Wallace**

**urn:lsid:zoobank.org:act:CDE21AE0-33FA-459D-ACD2-17A42796C04A**

*Myrmekiaphila torreya*, Gertsch and Wallace, 1936: 15. *Myrmekiaphila torreya*, Bond and Platnick 2007: 19–21.

*Molecular diagnosis*. Visual profile of sequence variation is summarized in Figure 6. All of the individual lineages within the *M. torreya* clade have unique diagnostic changes, however, the parsimony reconstruction only identifies a single change [C (965)] that has no homoplasy above the parent node for the “species”.

***Myrmekiaphila coreyi*** **Bond and Platnick**

**urn:lsid:zoobank.org:act:B611B216-BE0C-4622-AD13-F490128F0533**

*Myrmekiaphila coreyi* Bond and Platnick, 2007: 13, 14.

*Molecular diagnosis. Myrmekiaphila coreyi* can be diagnosed on the basis of the following combination of unique 12S/16S nucleotide substitutions: G (324), C 488, A (459). This combination represents unique, uniform changes in the parent node to *M. coreyi* and the sister lineage (pos. 324 & 488) plus the state of site 459 (uniquely derived G in the derived *M. torreya* lineage). Visual profile of sequence variation is summarized in Figure 6.

*Corrigendum*. Bond and Platnick [28] incorrectly attributed the type material of *M. coreyi* to the American Museum of Natural History collection. The type specimens and other material examined from the same series are deposited in the Florida State Collection of Arthropods.

### *The* fluviatilis *species group*

***Myrmekiaphila neilyoungi*** **Bond and Platnick**

**urn:lsid:zoobank.org:act:61104E26-98C4-458D-B4B5-A2A528A2F476**

*Myrmekiaphila neilyoungi* Bond and Platnick, 2007: 21–24.

*Molecular diagnosis. Myrmekiaphila neilyoungi* can be diagnosed on the basis of the following combination of unique 12S/16S nucleotide substitutions (given the number of changes along this very long branch we note only those changes that are unique and uniform for the lineage): *A (193), T (341), G (369), C (409), G (415), G (443), T (447), G (486), C (527), G (568), G (665), G (725), G (726), A (727), C (763), G (844), T (858), A (879), T (912), T (933), C (949), A (1094), A (1206), G (1253), C (1308)*. Visual profile of sequence variation is summarized in Figure 6.

***Myrmekiaphila foliata*** **Atkinson**

**urn:lsid:zoobank.org:act:F982C61C-95EF-463E-B0DE-518660E6A350**

*Myrmekiaphila foliata* Atkinson, 1886: 132; *Myrmeciophila atkinsoni* Simon, 1891: 316 *Myrmekiaphila fluviatilis* (misidentification): Bishop and Crosby, 1926: 166; *Myrmekiaphila foliata* Bond and Platnick, 2007: 9, 10.

*Molecular diagnosis. Myrmekiaphila foliata* can be diagnosed on the basis of the following unique combination of 12S/16S nucleotide substitutions: G (110), *T (118)*, T (119), *A (130)*, G (275), *C (290)*, T (368), T (373), T (375). Visual profile of sequence variation is summarized in Figure 6.

***Myrmekiaphila jenkinsi*** **Bond and Platnick**

**urn:lsid:zoobank.org:act:7AB0F182-493C-4378-B10C-26A9B6F717E9**

*Myrmekiaphila jenkinsi* Bond and Platnick, 2007: 16–19.

*Molecular diagnosis. Myrmekiaphila jenkinsi* can be diagnosed on the basis of the following combination of unique 12S/16S nucleotide substitutions (given the number of changes along this very long branch we note only those changes that are unique and uniform for the lineage): *T (100), T (159), C (240), C (375), T (421), G (473), T (539), A (548), T (562), C (568), T (732), C (758), T (839), A (968), G (1160), G (1298)*. Visual profile of sequence variation is summarized in Figure 6.

***Myrmekiaphila fluviatilis*** **(Hentz)**

**urn:lsid:amnh.org:spidersp:000513**

*Mygale fluviatilis* Hentz, 1850: 286; *Bolostromus fluviatilis* Banks, 1892: 147; *Myrmeciophila fluviatilis*, Banks, 1900: 530; *Myrmekiaphila fluviatilis*, Bond and Platnick, 2007: 14-16.

*Molecular diagnosis. Myrmekiaphila fluviatilis* can be diagnosed on the basis of the following unique combination of 12S/16S nucleotide substitutions: T (191), A (344), T (468), *T (557)*, *T (624)*, G (704), G (960), C (1006). Visual profile of sequence variation is summarized in Figure 6.

### *Species* incertae sedis

***Mymekiaphila flavipes*** **(Petrunkevitch)**

**urn:lsid:amnh.org:spidersp:000459**

*Aptostichus flavipes*, Petrunkevitch, 1925: 317; *Myrmekiphila flavipes*, Bond and Platnick, 2007: 29.

***Myrmekiaphila minuta*** **Bond and Platnick**

**urn:lsid:zoobank.org:act:04A8D838-A413-49E1-91B2-116D9AB68454**

*Myrmekiaphila minuta* Bond and Platnick, 2007: 27–29.

*Remarks*. Based on patterns in geography, morphology, and phylogeny of other species, we suspect that *M. minuta* will likely be placed into the *torreya* species group once molecular data become available.

*Corrigendum*. Bond and Platnick [28] incorrectly attributed the type material of *M.minuta* to the American Museum of Natural History collection. The type specimens and other material examined from the same series are deposited in the Florida State Collection of Arthropods.

## Materials and Methods

### Taxon Sampling

Every effort was made to sample all 11 species of *Myrmekiphila*. Following Bond and Stockman [14] we attempted to collect 2–3 individuals per population at localities where a species was common [23]. However, due to the rarity of some species fewer specimens were recovered. Each specimen was assigned a unique voucher number and haplotype designation; all specimens collected as part of this study will be deposited in the American Museum of Natural History and Field Museum of Natural History collections.

### Molecular Protocols

Protocols for obtaining and storing tissue samples and for performing DNA extractions are described in Hendrixson and Bond [66]. DNA amplification was preformed using the polymerase chain reaction (PCR) for two gene fragments [12S/16S mtDNA rRNA gene region and *glutamyl- & prolyl-tRNA synthetase* (192fin) nuclear protein coding region] for subsequent sequence analysis. 12S/16S mtDNA was amplified using the following PCR cocktail (50 µL final volume): 25 µL FailSafe PCR 2× Premix I (Epicentre, Madison, WI); 14.5 µL ultra pure water (Water Optima, Fisher Scientific, Hampton, NH); 5 µL of each 2.5 pM/µL primer; 0.5 µL *Taq* DAN polymerase (Invitrogen, Carlsbad, CA); and 1 µL genomic DNA. Primers LR-J-12887, SR-N-13xxxa, and SR-N-14612 [67] were used for amplification. Thermal cycle parameters were as follows: initial denaturation at 95°C for 2 min; 29 cycles of denaturation at 94°C for 30 s, annealing at 48°C for 30 s, and extension at 72°C for 1 min; and final extension at 72°C for 2 min. 192fin amplifications were carried out using GoTaq® Green Master Mix (Promega, Maddison, WI) with the primers 192fin_1F and 192fin_2R [68] under the following conditions: initial denaturation at 95°C for 5 min; 39 cycles of denaturation at 95°C for 30 s, annealing at 48°C for 30 s, and extension at 72°C for 1 min; and final extension at 72°C for 5 min. PCR products were verified on an agarose gel and purified using ExoSAP-IT (USB, Cleveland, OH).

Final purified PCR products were sequenced with an ABI Prism 3730 automated DNA sequencer (Applied Bio-systems, Foster City, CA) using the ABI Big Dye Terminator version 3.2 Cycle Sequencing Ready Reaction Kit. PCR primers for 12S/16S and 192fin were used in direct sequencing. These products were purified using Sephadex G-50 (Sigma-Aldrich, St. Louis, MO). All sequences were manually edited using the program Sequencher (ver. 4.1.2, Genecodes, Madison, WI).

### Multiple Sequence Alignment

Sequences were aligned using MUSCLE version 3.6 [69], [70] using default parameters, followed by minor adjustments in MESQUITE version 2.72 [71] to correct obvious problems. The alignment of 192fin was unambiguous due to the lack of length variation among taxa. The 12S/16S dataset was mostly unambiguous but required slight adjustments in some areas due to differences in length of secondary structure-related sequence.

### Phylogenetic Analyses

The program Kakusan 3 [72] was used to determine the appropriate model of DNA substitution by the Bayesian Information Criterion (BIC). Phylogenetic analyses of the data matrices were run independently and as separate partitions of a concatenated matrix. The 12S/16S dataset was further partitioned by 12S, tRNA-VAL, and 16S. The protein coding locus 192fin was partitioned by codon position, and separate models were chosen for each position. MrBayes ver. 3.1.2 [73], [74] was used to infer the phylogeny using the models of DNA substitution indicated by BIC. The 12S/16S, 192fin, and combined datasets each comprised four concurrent Markov Chain Monte Carlo (MCMC) chains run for 6,000,000 generations, 2,000,000 generations, and 10,000,000 generations, respectively. Trees were saved to file every 100 generations. Conservatively, topologies in the first 25% of the posterior distribution were discarded as burn-in following visual inspection in the program Tracer [75]. Clade posterior probabilities were computed from the remaining trees. The reported likelihood scores for all topologies post burn-in were computed using the “sump” command in MrBayes.

### Bayes Factor assessment of taxon monophyly

To test the monophyly of taxa that were recovered as paraphyletic or polyphyletic in the concatenated analysis, separate Bayesian analyses of the concatenated dataset were ran using the same model parameters in which the topology was constrained to force monophyly (prior probability  = 1.00 for the constrained group). Bayes Factors were computed by subtracting the harmonic mean of the -log likelihood of the posterior distribution of trees post burnin from the unconstrained analysis from that of the constrained analysis [(B_10_  = (Harmonic Mean –log Likelihood H_1_) – (Harmonic Mean –log Likelihood H_0_)] [76]. The resulting values provided strength of difference between the constrained and unconstrained trees with 10 or greater indicating strong support for the preferred hypothesis. This test was done for the polyphyletic species groups by constraining the *M*. *foliata* group (species with an unbranched embolus) and for the paraphyletic species *M*. *torreya* by forcing exclusivity of the species.

### Ancestral Character State Reconstruction

Ancestral character state reconstructions for the divided versus undivided embolus that previously defined species groups in the genus *Myrmekiaphila* were carried out in the program MESQUITE. A likelihood-based reconstruction of ancestral states was run under the Markov *k* model [77], [78] of character evolution. Ancestral character state optimizations were inferred from and mapped on to the tree derived from the concatenated data set analysis.
